# The hidden face of Duchenne (Neuro)Muscular Dystrophy. Preliminary evidence of social cognition impairment as a feature of the neuropsychological phenotype of DMD

**DOI:** 10.3389/fpsyg.2024.1504174

**Published:** 2025-01-06

**Authors:** S. Parravicini, C. A. Quaranta, M. I. Dainesi, A. Berardinelli

**Affiliations:** ^1^Department of Brain and Behavioral Sciences, University of Pavia, Pavia, Italy; ^2^Pediatric Neuroscience Unit, IRCCS Mondino Foundation, Pavia, Italy

**Keywords:** Duchenne Musclar Dystrophy, NEPSY II, social cognition, emotion recognition, pediatric, neuropsychological test

## Abstract

**Aim:**

To study unexplored neuropsychological domains in the characterization of the Central Nervous System (CNS) involvement in Duchenne Muscular Dystrophy (DMD) that could be relevant based on the recent findings about dystrophin expression in human CNS.

**Method:**

A sample of DMD individuals (*n* = 20) underwent a neuropsychological battery encompassing standard cognitive assessments but also less explored social cognition skills. Wechsler scales and a developmental neuropsychological assessment (NEPSY-II) were adopted.

**Results:**

Our sample performed significantly worse than the reference scores in the “social cognition” sub-items of the NEPSY-II; the difference persisted even when splitting the sample by Dp140 depletion or cognitive deficit. The difference in the “affect recognition” scores between the Dp140 + and the Dp140− subgroups was confirmed even after excluding the subjects with a cognitive deficit.

**Interpretation:**

Dystrophin is highly expressed in structures involved in the brain networks underlying some social cognition skills. Our results, which provide additional preliminary evidence of a possible specific impairment in this area, underscore the importance of considering social cognition as another feature of the CNS phenotype of DMD, consistent with a few other previous reports.

## Highlights

- In previous literature, social cognition was the least assessed neuropsychological domain.
- Evidence of social cognition impairment as part of the DMD’s neuropsychological phenotype.
- Study of unexplored features relevant in DMD based on dystrophin expression profile.

## Introduction

Duchenne Muscular Dystrophy (DMD) is the most common neuromuscular disorder with a pediatric onset. It is caused by mutations in the DMD gene on the X chromosome, which encodes for dystrophin. Its phenotype is characterized by progressive muscle degeneration with loss of muscular strength, leading to severe mobility limitations, including the loss of walking.

Furthermore, it has been described how individuals with DMD have a higher risk of manifesting neuropsychological impairments affecting cognitive functions (lower IQ compared to the general population), in particular, learning abilities (mainly reading) and executive functions such as information processing and working memory (Astrea et al., 2015; Doorenweerd, 2020). Furthermore, some neurodevelopmental and psychiatric disorders are frequently identified as comorbidities of DMD (Banihani et al., 2015; Battini et al., 2021; Hendriksen and Vles, 2008; Ricotti et al., 2016a, 2016b). These features have been increasingly reported as frequent manifestations of DMD, delineating Central Nervous System (CNS) involvement in dystrophinopathies.

Recent pathological studies have clarified the expression pattern of dystrophin in the CNS. In particular, the highest levels of dystrophin expression were found in sub-cortical structures such as the amygdala and hippocampus (Doorenweerd et al., 2018).

The amygdala’s involvement in various neurological pathologies (Genizi et al., 2012; Wang and Li, 2023) leads to a deficit in the emotional interpretation of facial expressions. This element is not reported in the classic clinical picture of DMD, and evidence of a deficit in this function was reported in a single paper (Hinton et al., 2007).

The current indications for diagnosis and management of DMD recommend the assessment of the neuropsychological profile at diagnosis. In particular, the adoption of standardized tests to assess children’s cognitive development, academic skills, social functioning, and emotional and behavioral regulation is suggested (Birnkrant et al., 2018).

The current literature highlighted how heterogeneously this recommendation was declined in clinical practice and research. Previous reviews reported the adoption of a wide variety of tools for assessing cognitive and neuropsychological profiles in DMD (Cotton et al., 2001; Hellebrekers et al., 2019) The multicentre study Brain INvolvement in Dystrophinopathies (BIND, n.d.) (including seven European neuromuscular centers from the UK, Denmark, France, Italy, Netherlands, and Spain) recently surveyed five of the seven participating centers to describe which tests are used in clinical practice for the assessment of the neuropsychological profile of individuals with DMD (Weerkamp et al., 2023). This study was representative of specialized centers’ expertise for neuromuscular disorders and extremely useful in understanding the topic’s complexity. The heterogeneity of tools used highlighted the complexity of the CNS-related phenotype of dystrophinopathies and the need for a shared and standardized tool kit to support comparative work.

A test battery, with a core set of clinical setting tools and other tests more suitable for research purposes, was outlined in the same paper. Such a tool kit would include general cognitive scales (i.e., Wechsler Intelligence Scales) and tests assessing the domains that were described as predominantly affected in dystrophinopathies (i.e., memory, attention, executive functioning, language, academics, ASD, ADHD, OCD, anxiety and depression) (Hendriksen et al., 2020; Snow et al., 2013; Pascual-Morena et al., 2023).

In this background, the assessment of social cognition skills is not widespread in clinical practice or research contexts. Among the tools classified in the BIND project survey, only a minority had the specific purpose of investigating these skills. In particular, a few tests designed to evaluate general neuropsychological functions (i.e., NEPSY-II) or specific pathologies (e.g., ADOS-2, ADI-R) include items dedicated to assessing social cognition.

Among them, NEPSY-II has been diffusely adopted to assess social cognition abilities such as the theory of mind (i.e., the ability to share another person’s perspective or mentally represent someone else’s intention) and affect recognition (i.e., the ability to perceive and interpret social cues to interpret the emotional meaning of others’ behavior) in other pediatric neuropsychiatric diseases.

Recently, one cross-sectional study tested a sample of DMD/BMD children and adolescents with a neuropsychological battery, including the Social Perception Domain of the NEPSY-II, the Reading the Mind in the Eyes Test, and the Strange Stories test (García et al., 2024). To the best of our knowledge, this is the first study, together with the paper by Hinton et al. (2007) mentioned above, to demonstrate an impairment in social cognition skills in dystrophinopathies.

The impact of CNS-related comorbidities on the quality of life for both individuals with DMD and their carers is becoming increasingly evident, especially considering the prolonged life expectancy due to the application of the current standard of care. During the 249th ENMC International Workshop, parents’ and patients’ associations further stressed this aspect by suggesting the idea of changing the label “DMD” into “DND” (i.e., Duchenne Neuromuscular Dystrophy) (Hendriksen et al., 2020). Therefore, the characterization of the CNS-related phenotype and the development of a Standard Operating Procedure (SOP) for assessing the neuropsychological profile in DMD is crucial.

Thus, the aim of this study is to provide further data about the possible impairment of social cognition skills in DMD children, identifying unexplored areas that could be relevant based on the recent findings about dystrophin expression in human CNS.

## Methods

We designed a cross-sectional study that included DMD individuals identified from the clinical database and the incident cases of the child neuropsychiatry department of a specialized center in northern Italy (IRCCS Mondino Foundation, Pavia, Italy). The subjects were selected based on the following inclusion criteria: male sex, availability of DMD genetic diagnosis data, age between 7 and 16 and 11 (end values included), and good knowledge of the Italian language. Subjects diagnosed with other concomitant genetic diseases were excluded from the study.

The inclusion in the study of all subjects is free and subject to the acquisition of the informed consent of the parents/legal representatives and—whenever possible—the subject’s consent. The study protocol was approved by the local Ethics Committee and was conducted according to the Declaration of Helsinki.

### Study protocol

Based on the available literature and the results of our review, we assembled a neuropsychological battery encompassing standard assessments (i.e., general cognitive profile, executive functions, memory, attention) but also less explored social cognition skills such as theory of mind and emotional recognition.

In particular, we adopted two standardized tests designed for a general cognitive and neuropsychological assessment:

- Wechsler cognitive scales: we adopted the Wechsler Intelligence Scale for Children IV (Orsini et al., 2012). All the items were administered to calculate the general IQ and the other indexes [Verbal Comprehension Index (VCI), Perceptual Reasoning Index (PRI), Working Memory Index (WMI), and Processing Speed Index (PSI)].
- NEPSY-II (Urgesi et al., 2011): selected tasks from the Attention and Executive Functioning (Visual Attention, Design Fluency, Auditory Attention and Response Set, Inhibition and Animal Sorting), Memory and Learning (Memory List), and Social Cognition (Theory of Mind and Affect Recognition) domains were adopted.

### Statistical planning and data analysis

At the time of the project conceptualization, no other studies had investigated social cognition in DMD samples. Therefore, the sample size and effect size were hypothesized based on preliminary data derived from the results of NEPSY-II adoption in a small group of DMD individuals (*n* = 11) during routine clinical assessment. In particular, the mean performance in the Social Cognition tasks (Theory of Mind and Affect Recognition) and the median standard score value were 5.00 (SD 3.95) and 4.60 (SD 2.95). We applied the results from both tasks to calculate the effect size, and we adopted the larger resulting sample size (i.e., the one derived from the preliminary results of the Theory of Mind task). The software we used was G * Power Version 3.1.9.6103,104, and an “*a priori*” analysis of the required sample size was performed (alpha error 0.05, power 0.95, one tail; considering an effect size d = 1.27).

A minimum necessary sample size of 11 individuals was obtained. As the procedures were not invasive and the assessments were planned to be included in the routine check-up program, we expected a minimum dropout. Thus, it was assumed to adopt an oversampling of 20% (minimum initial sample: 13 individuals).

The scores obtained from the administration of standardized tests were analyzed by an independent samples t-test (or non-parametric analog) to verify the presence of significant differences between the performance of DMD individuals and the normative data.

## Results

### Descriptives

All individuals were males, without cardiac-respiratory involvement. The overall general features of the sample are summarized in Table 1; the descriptive analysis of the sample split into two subgroups based on the theoretical depletion of the Dp140 isoform of dystrophin is also provided.

**Table 1:** Clinical overview of the sample: data about age, school attendance, age at symptoms onset/diagnosis, therapy, ambulation, and cardiac/respiratory function are summarized in the table.

	DMD	Dp140+	Dp140−
Numerosity	22 (100%)	9 (41%)	13 (59%)
Age [y]*	12.27 (3.07; 7–17)	13.33 (3.32; 7–17)	11.54 (2.79; 7–16)
Age at diagnosis [y]*	3.64 (1.71; 1–7)	3.22 (1.30; 1–5)	4.92 (1.94; 1–7)
Assuming corticosteroids**	95.5% (21/1)	89% (1/8)	100% (0/13)
Duration of the therapy with corticosteroids [m]*	95.48 (43.36; 17–184)	111.13 (52.55; 41–184)	85.85 (35.47; 17–143)
Ambulatory**	73% (16/4)	56% (5/4)	85% (11/2)

Tot = all included DMD individuals; Dp140+ = subgroup of DMD individuals with mutation theoretically not affecting the expression of Dp140; Dp140− = subgroup of DMD individuals with mutation theoretically affecting the expression of Dp140. *Mean (SD; range). ** % of individuals presenting the condition mentioned in the first column (n°pts presenting the condition/n° individuals not presenting the condition).

The mean overall IQ was 83.73, which aligns with the one-standard-deviation shift described in the literature for DMD individuals compared to the general population (see Table 2).

**Table 2:** Cognitive assessment: an overview of the scores obtained by administering Wechsler’s scales for general cognitive assessment.

Wechsler indexes	Tot (*n* = 20)	Dp140+ (*n* = 9)	Dp140− (*n* = 13)	Student’s *t*-test (*p*-value)
Full intellectual quotient*	83.73 (22.57; 40–127)	92.11 (17.04; 67–127)	77.92 (24.68; 40–115)	1.491 (0.152)
Verbal comprehension index*	93.50 (22.00; 54–126)	94.56 (19.91; 54–121)	92.77 (24.10; 56–126)	0.183 (0.857)
visual–spatial index*	95.77 (23.92; 48–139)	105.11 (16.68; 85–139)	89.31 (26.55; 48–128)	1.577 (0.131)
Working memory index*	73.32 (20.11; 46–123)	83.44 (23.46; 55–123)	66.31 (14.50; 46–85)	2.124 (0.046)
Processing speed index*	82.36 (15.06; 53–106)	90.22 (8.96; 74–103)	76.92 (16.28; 53–106)	2.218 (0.038)

Tot = all included DMD individuals; Dp140+ = subgroup of DMD individuals with mutation theoretically not affecting the expression of Dp140; Dp140− = subgroup of DMD individuals with mutation theoretically affecting the expression of Dp140. *Mean values (SD; range).

### Social cognition

We administered the sub-items “Theory of mind” and “Affect recognition” from the “Social Cognition” domain of NEPSY-II to our sample; two boys were not able to complete the assessment because of behavioral issues related to a comorbid neurodevelopmental disorders (one is a person with ASD and another has a cognitive deficit combined with emotional-behavioral dysregulation). Thus, the total number of subjects included in this analysis was 20.

**Figure 1:**
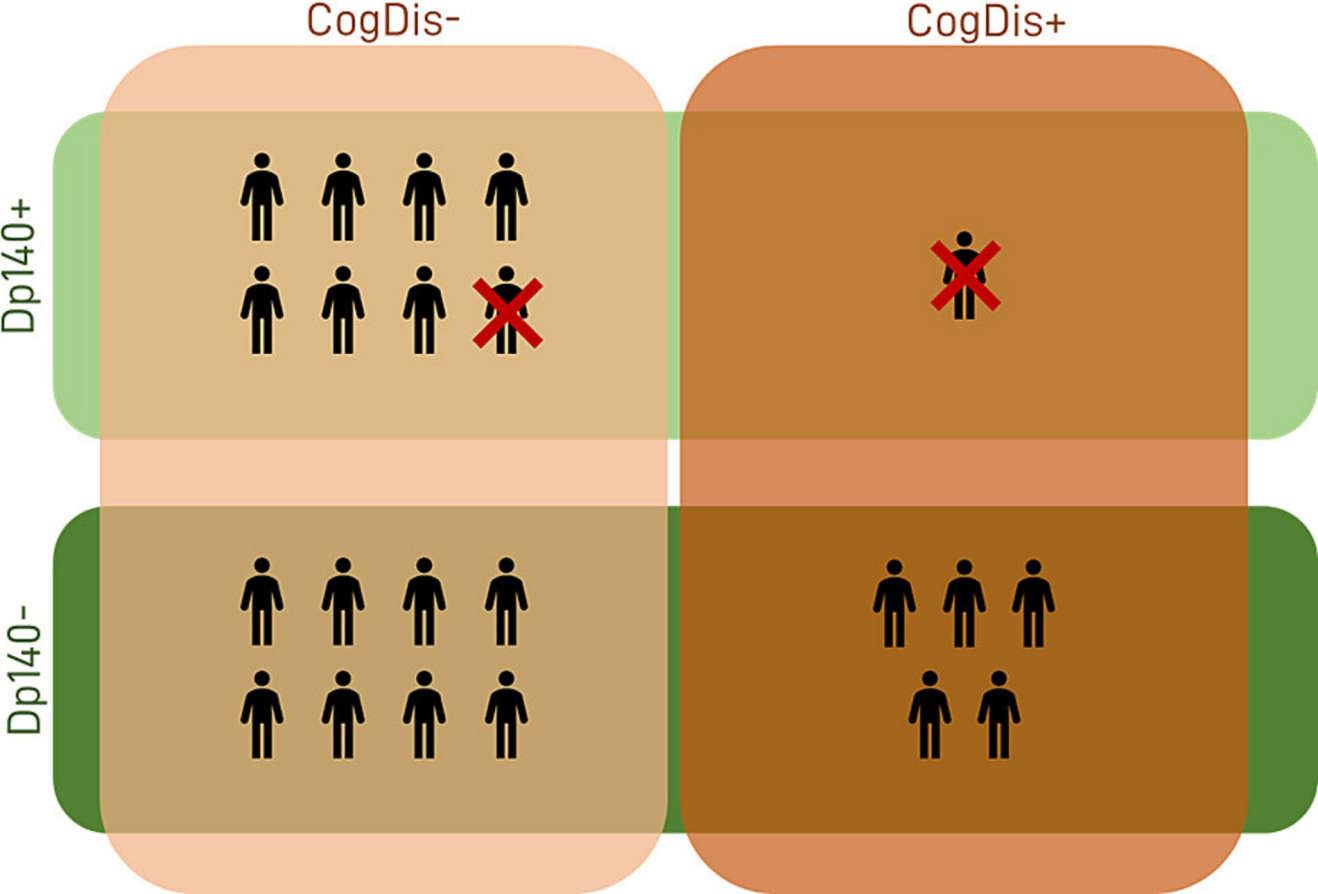
Sample descriptives. The features of our sample in terms of cognitive profile and expression of the Dp140 isoform of dystrophin are summarized in the figure. Dp140+ = subgroup of DMD individuals with mutation theoretically not affecting the expression of Dp140; Dp140− = subgroup of DMD individuals with mutation theoretically affecting the expression of Dp140; CogDis+ = subgroup of DMD individuals with IQ ≤ 70; CogDis− = subgroup of DMD individuals with IQ > 70.

Our sample size was limited, and a control group was not included. Thus, we analyzed the median standard scores and compared them to the reference score reported in the normative data of the test (i.e., 10). The overall scores obtained by our subjects were significantly lower than the reference in both sub-items; such difference was also confirmed when the sample was split based on the presence of Dp140 depletion (Dp140+ vs. Dp140−) or cognitive deficit (CogDis+ vs. CogDis−; threshold: total IQ ≤ 70) and the same analysis was singularly repeated on each subgroup (see Table 3; Figure 1).

**Table 3:** Social cognition—part 1: an overview of the standard scores obtained by administering the specific battery of the NEPSY-II scale.

NEPSY-II “social cognition” subtests	Tot	One sample *t*-test**	Dp 140+	One sample *t*-test**	Dp 140−	One sample *t*-test**	CogDis+	One sample *t*-test**	CogDis−	One sample *t*-test**
Number of subjects	20	***	7	***	13	***	5	***	15	***
Theory of mind*	5; 5.55 (3.99; 1–12)	13.5 (0.001)	5; 5.29 (4.50; 1–12)	2.500 (0.030)	5; 5.69 (3.88; 1–12)	5.000 (0.004)	2; 2.40 (1.67; 1–5)	0.000 (0.029)	6; 6.60 (4.01; 1–12)	13.500 (0.008)
Affect recognition*	5; 4.95 (3.35; 1–10)	0.000 (<0.001)	6; 7.00 (2.58; 4–10)	0.000 (0.029)	2; 3.85 (3.26; 1–10)	0.000 (0.001)	1; 1.20 (0.45; 1–2)	0.000 (0.024)	6; 6.20 (2.91; 1–10)	0.000 (0.001)

The standard scores obtained by DMD were tested for differences from the reference scores (test value: 10) by a non-parametric test due to a deviation from the assumption of normality (Shapiro–Wilk test). Tot = all included DMD individuals; Dp140+ = subgroup of DMD individuals with mutation theoretically not affecting the expression of Dp140; Dp140− = subgroup of DMD individuals with mutation theoretically affecting the expression of Dp140; CogDis + = subgroup of DMD individuals with general IQ ≤ 70at the Wechsler scale; CogDis− = subgroup of DMD individuals with general IQ > 70 at the Wechsler scale. For all tests, the alternative hypothesis specifies that the median is less than 10. *Median; mean standard scores (SD; range). **Wilcoxon signed-rank test (*p*-value).

**Figure 2:**
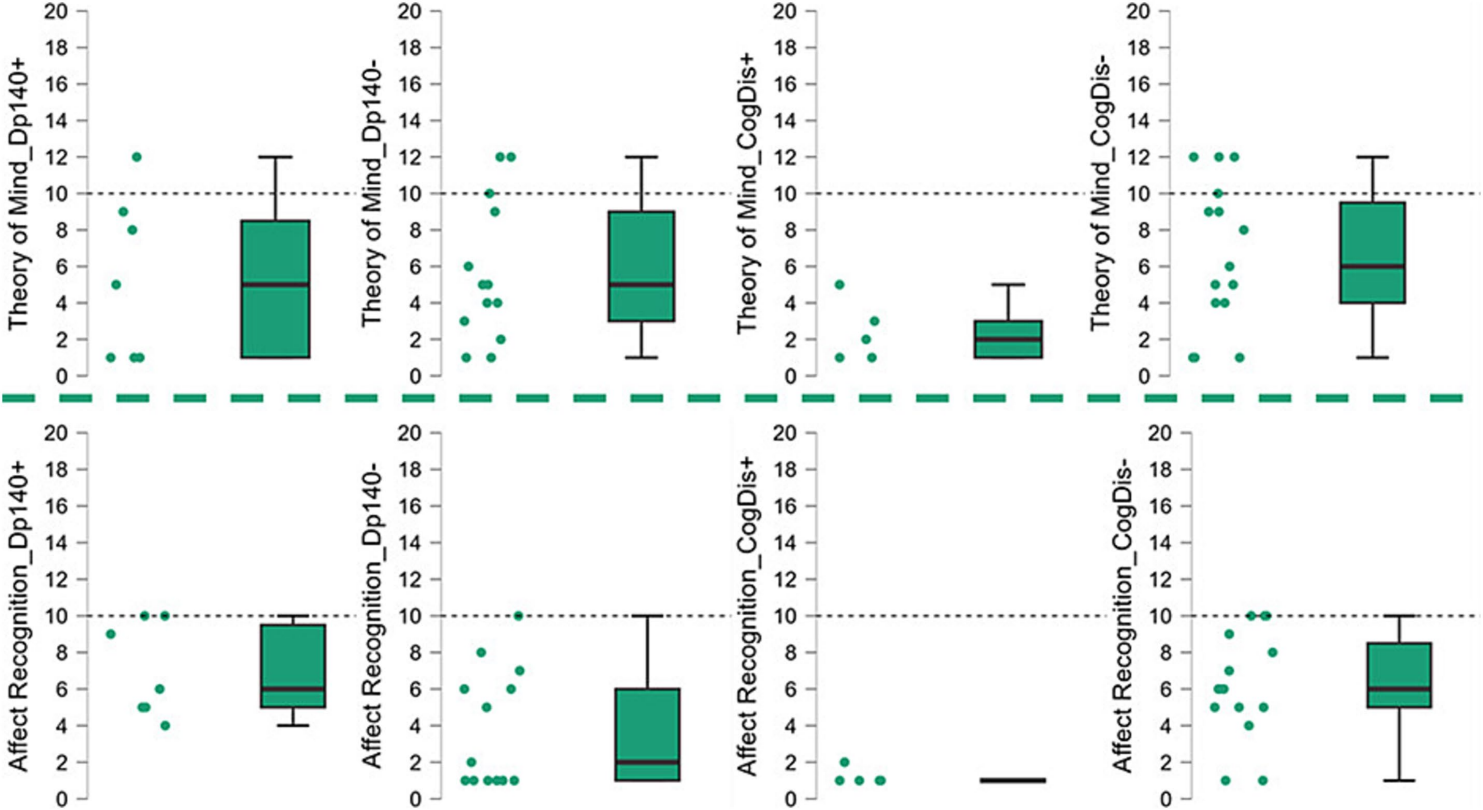
Social cognition—part 1. The graphs refer to the data shown in Table 3 and represent the performance of our sample in the “social cognition” items of the NEPSY-II scale, showing the data dispersion and the differences between the standard scores from the reference value (=10). Dp140+ = subgroup of DMD individuals with mutation theoretically not affecting the expression of Dp140; Dp140− = subgroup of DMD individuals with mutation theoretically affecting the expression of Dp140; CogDis + = subgroup of DMD individuals with general IQ ≤ 70at the Wechsler scale; CogDis− = subgroup of DMD individuals with general IQ > 70 at the Wechsler scale.

Then, the differences between each couple of subgroups were tested to explore the role of the cognitive deficit and the expression of the Dp140 dystrophin isoform. The only significant difference emerged in the “affect recognition” sub-item, with the subset of individuals with cognitive deficit scoring worse than those without it. Nevertheless, it is noteworthy that a similar difference in the “affect recognition” scores also emerged between the Dp140+ (better) and the Dp140− (worse) subgroups, with a *p*-value close to significance (see Table 4; Figure 2).

**Table 4:** Social cognition—part 2: an overview of the differences in standard scores obtained by administering the specific battery of the NEPSY-II scale between subgroups based on the presence/absence of Dp140 depletion and cognitive deficit.

NEPSY-II “social cognition” subtests	Tot	Dp140+	Dp140−	Independent samples *t*-test**	CogDis+	CogDis−	Independent samples *t*-test**
Number of subjects	20	7	13	***	5	15	***
Theory of mind*	5; 5.55 (3.99 1–12)	5; 5.29 (4.50; 1–12)	5; 5.69 (3.88; 1–12)	40.500 (0.718)	2; 2.40 (1.67; 1–5)	6; 6.60 (4.01; 1–12)	60.000 (0.052)
Affect recognition*	5; 4.95 (3.35; 1–10)	6; 7.00 (2.58; 4–10)	2; 3.85 (3.26; 1–10)	68.000 (0.076)	1; 1.20 (0.45; 1–2)	6; 6.20 (2.91; 1–10)	69.000 (0.006)

The standard scores were tested for differences by a non-parametric test due to deviation from the assumption of normality (Shapiro–Wilk test). Tot = all included DMD individuals; Dp140+ = subgroup of DMD individuals with mutation theoretically not affecting the expression of Dp140; Dp140− = subgroup of DMD individuals with mutation theoretically affecting the expression of Dp140; Cognitive Deficit + = subgroup of DMD individuals with general IQ ≤ 70at the Wechsler scale; Cognitive Deficit − = subgroup of DMD individuals with general IQ > 70 at the Wechsler scale. *Median standard scores; mean standard scores (SD; range). ** Mann–Whitney U test (*p*-value).

**Figure 3:**
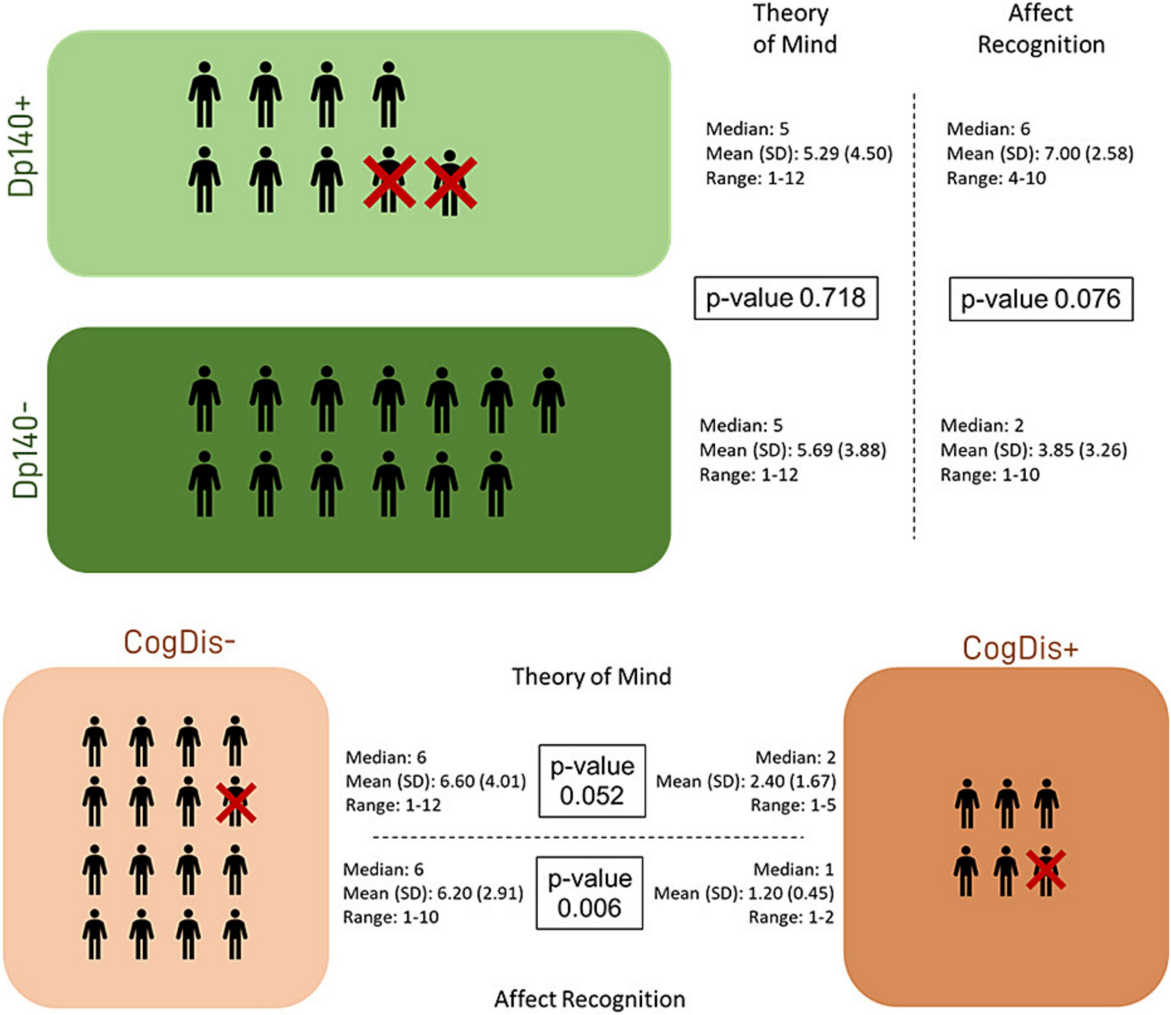
Social cognition—part 2. The figure refers to the data of Table 4. It represents the differences in standard scores obtained in our sample by administering the specific battery of the NEPSY-II scale. The differences were tested between subgroups based on the presence/absence of Dp140 depletion and cognitive deficit. Dp140+ = subgroup of DMD individuals with mutation theoretically not affecting the expression of Dp140; Dp140− = subgroup of DMD individuals with mutation theoretically affecting the expression of Dp140; CogDis + = subgroup of DMD individuals with general IQ ≤ 70 at the Wechsler scale; CogDis− = subgroup of DMD individuals with general IQ > 70 at the Wechsler scale.

**Figure 4:**
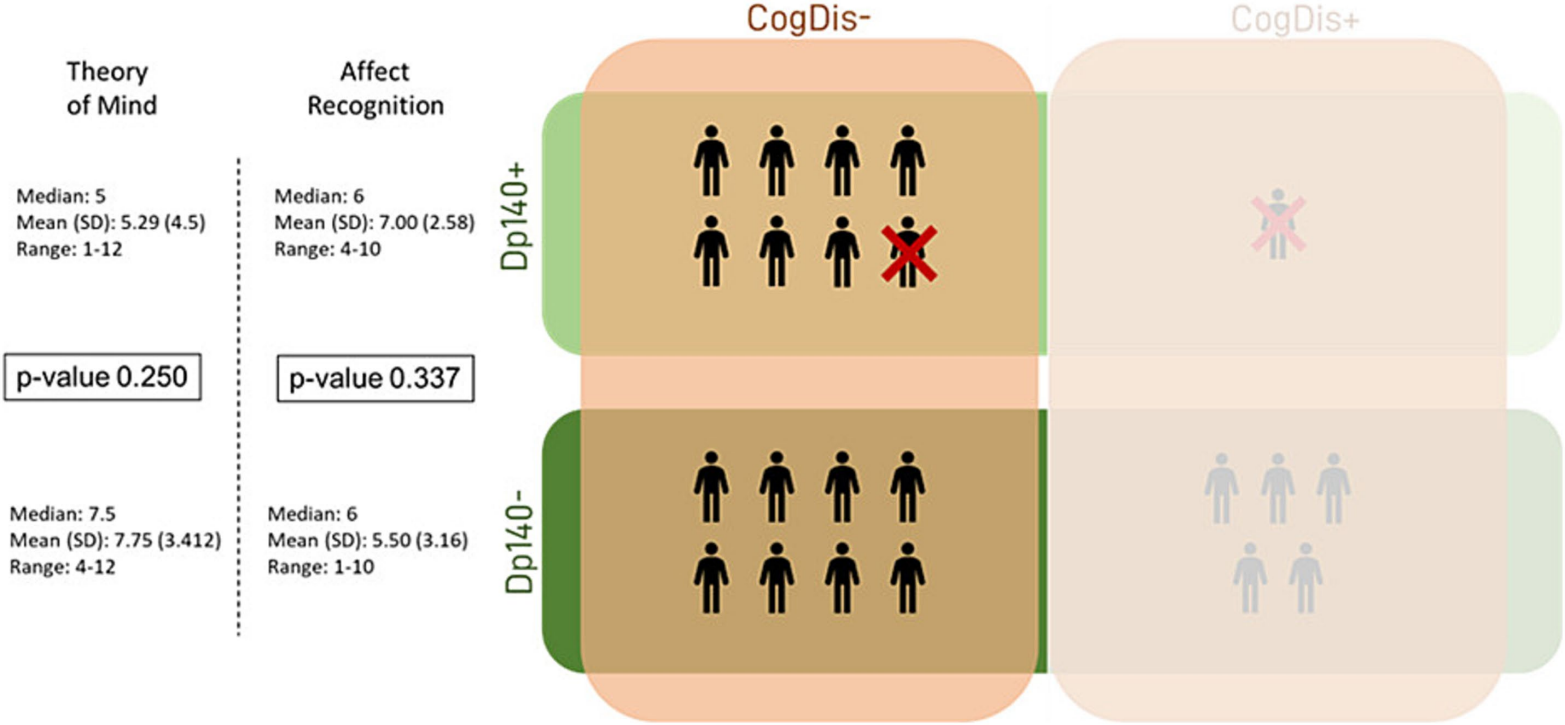
Social cognition—part 3. This figure refers to the data in Table 5. It represents the differences in standard scores obtained in our sample by administering the specific battery of the NEPSY-II scale. The differences were tested between subgroups based on the presence/absence of Dp140 depletion and cognitive deficit. Dp140+ = subgroup of DMD individuals with mutation theoretically not affecting the expression of Dp140; Dp140− = subgroup of DMD individuals with mutation theoretically affecting the expression of Dp140; CogDis + = subgroup of DMD individuals with general IQ ≤ 70 at the Wechsler scale; CogDis− = subgroup of DMD individuals with general IQ > 70 at the Wechsler scale.

Lastly, the same analysis was applied to the subset of subjects with total IQ > 70 (CogDis−) to assess the role of the expression of the Dp140 dystrophin isoform without the possible confounding effect of cognitive disability. No significant differences emerged, but interestingly, the divergence between the Dp140 + and the Dp140− subgroups in the “affect recognition” scores aligned with the results of the previous analysis, even excluding the subjects with a cognitive deficit, with a worse performance in the Dp140− subgroup (see Table 5; Figures 3, 4).

**Table 5:** Social cognition—part 3: an overview of the differences in standard scores obtained by the administration of the specific battery of the NEPSY-II scale between subgroups based on the presence/absence of Dp140 depletion (only subjects without cognitive deficit, i.e., IQ > 70).

NEPSY-II “social cognition” subtests	Tot	Dp140+	Dp140−	Independent samples *t*-test**
Number of subjects	15	7	8	***
Theory of mind*	6; 6.60 (4.014; 1–12)	5; 5.286 (4.499; 1–12)	7.50; 7.75 (3.412; 4–12)	−1.205 (0.250)
Affect recognition*	6; 6.20 (2.908; 1–10)	6; 7.00 (2.582; 4–10)	6; 5.50 (3.162; 1–10)	0.996 (0.337)

Tot = all included DMD individuals; Dp140+ = subgroup of DMD individuals with mutation theoretically not affecting the expression of Dp140; Dp140− = subgroup of DMD individuals with mutation theoretically affecting the expression of Dp140. *Median standard scores; mean standard scores (SD; range). ** Student’s *t*-test (*p*-value).

A high and significant positive correlation emerged between the Affect Recognition scores and VSI, WMI and PSI; the same kind of correlation was found between Affect Recognition Scores and general IQ (Pearson’s correlation tests, one-tailed for positive correlations: all Pearson *r* > 0.6 and all *p*-values<0.05).

## Discussion

Neuropsychological batteries in the evaluation of DMD are very heterogeneous, and the most commonly adopted tools are Wechsler scales and tests assessing the functions traditionally described as impaired in DMD. Thus, the neuropsychological domains mainly assessed were language, visual–spatial skills, memory, attention, and executive functioning.

Social cognition skills are rarely included in the neuropsychological assessment of DMD in clinical practice or research settings.

With this background, we designed a clinical protocol that was, to our knowledge, one of the first studies investigating this domain in DMD. Interestingly, our social cognition assessment yielded intriguing results, in line with those recently published in a similar experimental setting (García et al., 2024). Compared to the standard data, we consistently observed poor performances in our sample in the tasks assessing the theory of mind and recognizing the affective meaning of facial expressions.

In particular, among the social cognition skills we investigated, the impairment of affect recognition appeared more evident in our sample when comparing Dp140+ and Dp140− groups. This finding is preliminary and should be taken with caution, given the low numerosity and the absence of significance when analyzing without the influence of cognitive disability. Moreover, the high correlation between Affect Recognition scores and the cognitive indexes (total IQ, VSI, WMI and PSI) reflects the relationship between cognitive functioning and social cognition skills. Nevertheless, it provides additional evidence of a possible specific impairment in this area as another feature of the CNS phenotype of DMD, consistent with a few other previous reports (Hinton et al., 2007; García et al., 2024).

Previous studies have not largely explored this area, and there is no clinical evidence of such an impairment (except for the increased risk of comorbidity with ASD in DMD). However, the recent data from the Human Brain Atlas pointed out the high levels of dystrophin expression in the amygdala and hippocampus. These structures are involved in the brain networks underlying the social functions (e.g., the extra-geniculostriatal network) (De Gelder et al., 2011).

A common consideration about these hypotheses should be made. Various neural networks underlie all the mentioned functions, sometimes overlapping and involving the same CNS structures. Based mainly on functional neuroimaging, it has been possible to identify the brain regions involved in executive (involving the prefrontal and parietal cortex, basal ganglia, thalamus, and cerebellum) and social functioning (amygdala, portions of occipital-temporal-frontal cortex, the Default Mode Network). Furthermore, these deficits are common to many neuropsychiatric diseases. Consequently, identifying the exact cause of an impairment widely involving these functions may not be obvious. For example, the hypothesis of cerebellar dysfunction was supported by clinical features (e.g., deficit in cognitive functions or memory) and evidence from animal models. Still, it was reconsidered based on analyzing dystrophin expression in the human brain. Nevertheless, the involvement of the same networks at another level remains plausible.

Future development of these preliminary findings might involve designing an experimental setting to specifically explore the behavioral correlates of the involvement of those CNS networks, including the subcortical structures characterized by a higher dystrophin expression.

## Data Availability

The datasets presented in this study can be found in online repositories. The names of the repository/repositories and accession number(s) can be found below: Zenodo (doi: 10.5281/zenodo.14415673).

## References

[ref1] Astrea G. Pecini C. Gasperini F. Brisca G. Scutifero M. Bruno C.. (2015). Reading impairment in Duchenne muscular dystrophy: a pilot study to investigate similarities and differences with developmental dyslexia. Res. Dev. Disabil. 45-46, 168–177. doi: 10.1016/j.ridd.2015.07.025, PMID: 26255617

[ref2] Banihani R. Smile S. Yoon G. Dupuis A. Mosleh M. Snider A.. (2015). Cognitive and neurobehavioral profile in boys with duchenne muscular dystrophy. J. Child. Neurol. 30, 1472–1482. doi: 10.1177/0883073815570154, PMID: 25660133

[ref3] Battini R. Lenzi S. Lucibello S. Chieffo D. Moriconi F. Cristofani P.. (2021). Longitudinal data of neuropsychological profile in a cohort of Duchenne muscular dystrophy boys without cognitive impairment. Neuromuscul. Disord. 31, 319–327. doi: 10.1016/j.nmd.2021.01.011 33658162

[ref4] BIND. https://bindproject.eu/. BIND, Horizon 2020–847826.

[ref5] Birnkrant D. J. Bushby K. Bann C. M. Alman B. A. Apkon S. D. Blackwell A.. (2018). Diagnosis and management of Duchenne muscular dystrophy, part 2: respiratory, cardiac, bone health, and orthopaedic management. Lancet Neurol. 17, 347–361. doi: 10.1016/S1474-4422(18)30025-5, PMID: 29395990 PMC5889091

[ref6] Cotton S. Voudouris N. J. Greenwood K. M. (2001). Intelligence and Duchenne muscular dystrophy: full-scale, verbal, and performance intelligence quotients. Dev. Med. Child Neurol. 43, 497–501. doi: 10.1017/s0012162201000913, PMID: 11463183

[ref7] De Gelder B. Van Honk J. Tamietto M. (2011). Emotion in the brain: of low roads, high roads and roads less travelled. Nat. Rev. Neurosci. 12:425. doi: 10.1038/nrn2920-c1, PMID: 21673722

[ref8] Doorenweerd N. (2020). Combining genetics, neuropsychology and neuroimaging to improve understanding of brain involvement in Duchenne muscular dystrophy—a narrative review. Neuromuscul. Disord. 30, 437–442. doi: 10.1016/j.nmd.2020.05.001, PMID: 32522501

[ref9] Doorenweerd N. Mahfouz A. Van Putten M. Kaliyaperumal R. T’Hoen P. A. C. Hendriksen J. G. M. M.. (2018). Timing and localization of […] dystrophy. Sci Rep. 2017 Oct 3;7(1):12575. doi: 10.1038/s41598-017-12981-5. Erratum in: Sci. Rep. 8:4058. doi: 10.1038/s41598-018-22154-7, PMID: 28974727 PMC5626779

[ref10] García I. Martínez O. López-Paz J. F. García M. Espinosa-Blanco P. Rodríguez A. A.. (2024). Social cognition in DMD and BMD dystrophinopathies: a cross-sectional preliminary study. Clin. Neuropsychol. 38, 219–234. doi: 10.1080/13854046.2023.2202332, PMID: 37081823

[ref11] Genizi J. Shamay-Tsoory S. G. Shahar E. Yaniv S. Aharon-Perez J. (2012). Impaired social behavior in children with benign childhood epilepsy with centrotemporal spikes. J. Child. Neurol. 27, 156–161. doi: 10.1177/0883073811414420, PMID: 21868370

[ref12] Hellebrekers D. M. J. Lionarons J. M. Faber C. G. Klinkenberg S. Vles J. S. H. JGM H. (2019). Instruments for the assessment of behavioral and psychosocial functioning in Duchenne and Becker muscular dystrophy; a systematic review of the literature. J. Pediatric Psychol. 44, 1205–1223. doi: 10.1093/jpepsy/jsz062, PMID: 31429914

[ref13] Hendriksen J. G. M. Thangarajh M. Kan H. E. Muntoni F. Aoki D. Y. Collin D. P.. (2020). 249th ENMC international workshop: the role of brain dystrophin in muscular dystrophy: implications for clinical care and translational research, Hoofddorp, the Netherlands, November 29th–December 1st 2019. Neuromuscul. Disord. 30, 782–794. doi: 10.1016/j.nmd.2020.08.357, PMID: 32912717

[ref14] Hendriksen J. G. M. Vles J. S. H. (2008). Neuropsychiatric disorders in males with duchenne muscular dystrophy: frequency rate of attention-deficit hyperactivity disorder (ADHD), autism spectrum disorder, and obsessive-compulsive disorder. J. Child. Neurol. 23, 477–481. doi: 10.1177/0883073807309775, PMID: 18354150

[ref15] Hinton V. J. Fee R. J. De Vivo D. C. Goldstein E. (2007). Poor facial affect recognition among boys with Duchenne muscular dystrophy. J. Autism. Dev. Disord. 37, 1925–1933. doi: 10.1007/s10803-006-0325-5, PMID: 17177118 PMC2084467

[ref16] Orsini A. Pezzuti L. Picone L. (2012). Wechsler intelligence scale for children IV-Edizione Italiana. Milano, Italy: Giunti O.S.

[ref17] Pascual-Morena C. Cavero-Redondo I. Martínez-Vizcaíno V. Sequí-Domínguez I. Fernández-Bravo-Rodrigo J. Jiménez-López E. (2023). Dystrophin genotype and risk of neuropsychiatric disorders in Dystrophinopathies: a systematic review and Meta-analysis. J. Neuromusc. Dis. 10, 159–172. doi: 10.3233/JND-221586 PMC10041431 36565132

[ref18] Ricotti V. Jägle H. Theodorou M. Moore A. T. Muntoni F. Thompson D. A. (2016a). Ocular and neurodevelopmental features of Duchenne muscular dystrophy: a signature of dystrophin function in the central nervous system. Europ. J. Hum. Genet. 24, 562–568. doi: 10.1038/ejhg.2015.135, PMID: 26081639 PMC4929863

[ref19] Ricotti V. Mandy W. P. L. Scoto M. Pane M. Deconinck N. Messina S.. (2016b). Neurodevelopmental, emotional, and behavioural problems in Duchenne muscular dystrophy in relation to underlying dystrophin gene mutations. Dev. Med. Child Neurol. 58, 77–84. doi: 10.1111/dmcn.12922, PMID: 26365034

[ref20] Snow W. M. Anderson J. E. Jakobson L. S. (2013). Neuropsychological and neurobehavioral functioning in Duchenne muscular dystrophy: a review. Neurosci. Biobehav. Rev. 37, 743–752. doi: 10.1016/j.neubiorev.2013.03.016, PMID: 23545331

[ref21] Urgesi C. Campanella F. Fabbro F. (2011). NEPSY-II, Contributo alla Taratura Italiana. Milano, Italy: Giunti O.S.

[ref22] Wang S. Li X. (2023). A revisit of the amygdala theory of autism: twenty years after. Neuropsychologia 183:108519. doi: 10.1016/j.neuropsychologia.2023.108519, PMID: 36803966 PMC10824605

[ref23] Weerkamp P. Chieffo D. Collin P. Moriconi F. Papageorgiou A. Vainieri I.. (2023). Psychological test usage in duchenne muscular dystrophy: an EU multi-Centre study. Eur. J. Paediatr. Neurol. 46, 42–47. doi: 10.1016/j.ejpn.2023.06.007, PMID: 37423006

